# Impact of virus-mediated bacterial interactions on acute gastroenteritis symptoms: A new scoring system for clinical assessment

**DOI:** 10.1080/21505594.2025.2529442

**Published:** 2025-07-07

**Authors:** Zhangkai Xu, Zishu Liu, Yuxiang Zhao, Jiang Chen, Weibo Cui, Wenjing Wan, Zhendi Yu, Qingyi Shao, Youshi Liu, Baolan Hu, Dongqing Cheng

**Affiliations:** a School of Medical Technology and Information Engineering, Zhejiang Chinese Medical University, Hangzhou, China; b Department of Clinical Laboratory, Zhejiang Hospital, Hangzhou, China; c College of Environmental and Resource Sciences, Zhejiang University, Hangzhou, China; d Department of Laboratory Medicine, The First Affiliated Hospital, Zhejiang University School of Medicine, Hangzhou, China; e Department of Laboratory Medicine, Zhejiang Provincial People’s Hospital Zhedong South Campus, Taizhou, China; f The Fourth Clinical Medical College, Zhejiang Chinese Medical University, Hangzhou, China

**Keywords:** Viral acute gastroenteritis, clinical symptom scoring scale, gut microbiota, virus-mediated bacterial interactions, *Prevotella/Bacteroides* index

## Abstract

Acute gastroenteritis (AGE) exerts a substantial healthcare burden and economic loss annually, mainly due to viral infections. The objective of the study was to elucidate the impact of the interactions between the AGE virus and gut microbiota on patient clinical symptoms, thereby facilitating the early diagnosis and treatment of AGE. Clinical information and fecal samples were collected from 289 AGE patients (exclude fungal, parasitic, and bacterial infections), of whom 23.5% were infected with AGE viruses. A scoring method was developed to assess the severity of virus-induced AGE in patients. The results indicate significant differences (*p* < 0.05 indicates a significant difference, as determined by Kruskal–Wallis test, *p* = 0.03) in clinical symptom scores among the None-virus, Single-virus, and Dual-virus group. The Single-virus (14.82) and Dual-virus (15.33) groups exhibited more severe clinical symptom, with scoring values higher than None-virus group (12.40). Although significant differences in microbial community composition were observed between the Single-virus and Dual-virus groups (as determined by Adonis analysis, Variation = 0.11, *p* = 0.034), the diversity index (e.g. Chao1) did not significantly differ among the None-virus (288.14), Single-virus (345.74), and Dual-virus (282.70) groups. Notably, the patients with a higher *Prevotella*/*Bacteroide*s index displayed more severe clinical symptom, as the index in the Single-virus and Dual-virus groups was over 10-times greater than in the None-virus group. In summary, this study shows that clinical symptoms of patients with viral AGE could be exacerbated through promoting bacterial competitions, and this understanding would facilitate the early diagnosis and treatment of viral AGE.

## Introduction

Acute gastroenteritis (AGE) is the second most burdensome infectious disease globally [1]. It causes approximately 1.3 million deaths globally every year and is one of the leading causes of mortality across all age groups [2], especially affecting the elderly, infants, children or immune-compromised individuals [3,4]. AGE is characterized by watery diarrhea or vomiting that occurs three or more times within a 24-h period [5–7]. This can be accompanied by low-grade fever or abdominal pain [3,8]. Patients may exhibit various clinical manifestations, including elevated white blood cell counts and C-reactive protein (CRP) levels, as well as the presence of red and white blood cells in the feces [9,10]. Viral AGE is usually self-limiting as there is no specific medication for it and the clinical symptoms may last less than a week [11]. However, failure to consider the onset and progression of the numerous symptoms associated with the disease may result in dehydration or, in severe cases, even death [3,7,11].

Viruses are the predominant pathogens responsible for AGE worldwide [12]. They have been identified in more than 55% of pathogens detected in patients with AGE [13], with the incidence rising to 80% among children under the age of five [14,15]. Viral AGE exhibits a high transmission capability through contaminated food, water, or virus-carrying excreta [16]. The major causative viruses include Norovirus (NoV), Rotavirus (RoV), and enteric adenovirus (EAdV) [17–19]. The AGE viruses are highly infectious, requiring only a few dozen viral particles to cause an infection [20]. These factors facilitate the spread of AGE viruses, leading to widespread infections and posing a significant threat to public health and safety in society [21].

The gut microbiota has been implicated in many diseases, particularly infectious diseases [22], but its role in virus-mediated AGE remains poorly understood. The gut microbiota forms complex communities, widely recognized as a primary factor regulating host health [23,24]. It maintains basic functions such as intestinal health and homeostasis and plays an essential role in pathophysiological processes [22,25]. One well-known function of the human gut microbiota is serving as the primary barrier to viral invasion, with commensal microbes playing a critical role in defending against viral infections [26,27]. For example, gut commensal bacteria *Bacteroidetes* can stimulate IFN-β through outer membrane-associated glycolipids, whose expression was involved in various immune responses against viral infections [26]. The gut-isolated bacterium *Limosilactobacillus fermentum* has been shown to significantly decrease the titer of NoV infection and acts as an antagonist against viral infections [28]. In contrast, it has been discovered that the cohabitation of viruses and particular bacteria could promote viral infection and negatively impact host health [24]. For instance, *Enterobacter cloacae* isolated from the human gut was reported to promote NoV infection of host cells [29,30]. Upon treating the gut microbiota of mice with antibiotics, RoV infectivity in mice declined by over 40%, and virus infection onset was delayed [31]. Furthermore, AGE virus infections may lead to prolonged destruction of the gut microbiota, and changes to the gut microbiota could increase the likelihood of gastrointestinal-related sequelae [32,33].

Viruses and gut microbiota frequently coexist and may have a dynamic interaction with each other [34,35]. However, the interplay between these factors and their impact on host health has rarely been documented [27,36]. Most studies have neglected the relationship between gut microbiota and the AGE viruses infecting patients, but exploring the uncertain relationship between the two, whether facilitating or inhibiting, has important implications for the health of AGE patients. Elucidating the intricate correlation between enteric viruses and gut bacterial community could serve as a valuable tool for the early detection and treatment of viral AGE.

Current research tends to focus on changes in the composition of the gut microbiota due to viral infections, while the impact of changes in the gut microbiota on patient health remains uncertain and understudied. In this study, 289 fecal samples from AGE patients were collected to establish clinical symptom score scale to assess the severity of clinical symptoms. Fecal samples with confirmed viral AGE were selected for high-throughput sequencing, and the correlation between the virus and the patient gut microbiota was analyzed by microbiome analysis. The relationship between the virus and the gut microbiota was investigated for its impact on the health of patients based on clinical symptom scores, with the aim of contributing to the early diagnosis and treatment of AGE patients.

## Materials and methods

### Collection of clinical samples

Two hundred and eighty-nine fecal samples from AGE patients were collected sequentially at Zhejiang Provincial People’s Hospital Zhedong South Campus in Taizhou, Zhejiang Province, China, Zhejiang Hospital and Hangzhou First People’s Hospital in Hangzhou, Zhejiang Province, China, during February 2021 and January 2022 for fecal routine and bacteriology examinations to exclude fungal, parasitic, and bacterial infections (i.e. *Salmonella* spp., *Shigella* spp., *Vibrio cholerae* and *Vibrio parahaemolyticus, and* Diarrheogenic *Escherichia coli*). Blood samples from AGE patients were collected for blood routine examination, and the associated clinical information was also gathered. AGE was defined by more than three times of diarrhea or vomiting within 24-h period. Patients were excluded from this study based on the following criteria: (1) those who had undergone surgery for various gastrointestinal conditions; (2) patients with other diseases, including immunodeficiency patients with major illnesses; and (3) patients receiving antibiotic or antiviral therapy [5–7]. All fecal samples were stored at −80°C for subsequent experiments. Detailed information about the samples was provided in the “Patients Summary” sheet in Supplementary Material S1, and the general characteristics of AGE patients are presented in Table S2.1 of Supplementary Material S2.

### Detection of AGE virus

The AGE viruses (Norovirus, Rotavirus, and Human enteric adenovirus) were detected by reverse transcription real-time fluorescence quantitative PCR (RT-qPCR). The qPCR reaction primer sequences were sourced from previous studies conducted by Yu et al. [37]. The primers were synthesized by Sangon Biotech (Shanghai, China). Positive control samples donated by the Zhejiang Provincial Center for Disease Control and Prevention. Further instructions on qPCR assay for AGE virus are detailed in Supplementary Material S3 (The specific primers and probes are shown in Table S3.1, and the PCR assay mixtures are shown in Tables S3.2 and S3.3), and the samples detection results are presented in Table S4.1 and S4.2 of Supplementary Material S4.

### Establishment of clinical symptom scoring scale for AGE patients

To establish a clinical symptom scoring scale for AGE patients, we included multiple clinical symptoms with reference to the modified Vesikari scale of Freedman [38]. The Vesikari scale considers the duration of diarrhea or vomiting and maximum rectal temperature as significant factors. However, since AGE patients are typically not hospitalized, the duration of their diarrhea or vomiting relies on self-reporting, introducing subjectivity into the recording of onset times, which may lead to inaccuracies. Additionally, most temperature measurements are taken using electronic thermometer for ear or forehead temperatures, mercury thermometer for axillary or oral temperature. Thus, the practicality of measuring the maximum rectal temperature is challenging and it is also difficult to ascertain the peak temperature. Therefore, these items have been omitted or modified in our clinical symptom scoring scale. To enhance the objectivity of the clinical symptom scoring scale, more objective examination indicators, such as stool and blood routine examinations, have been added. Patient indicators like diarrhea, vomiting, abdominal pain, and fever were included based on the modified Vesikari scale [38], fecal indicators such as fecal character, fecal occult blood, and fecal leukocytes were included with reference to Lai et al. [39], and inflammatory indicators such as white blood cell count, lymphocyte percentage, and CRP were included with reference to Zaraket et al. [40]. The clinical symptom scale was developed to provide an objective evaluation of clinical symptom from three perspectives: patients self-reporting, fecal status, and blood tests.

The AGE patients were categorized into three groups based on qPCR detection results of fecal samples: the None-virus group, the Single-virus group, and the Dual-virus group. Among the fecal samples from patients with single-virus infections (Single-virus), there were three subgroups: the Adenovirus group, the Norovirus group, and the Rotavirus group. The clinical symptom scoring method was objectively applied to evaluate each group with AGE virus infection.

### High-throughput amplicon sequencing analysis

Fecal samples were selected and sent to Magigene Biological Technology Co. Ltd. (Guangdong, China) for high-throughput sequencing. The basic information of the sequenced samples was presented in Table S5.1 of Supplementary Material S5. The bacterial 16S rRNA gene was amplified and sequenced using primers 515F-806 R for V4 region with the Illumina MiSeq platform, respectively. The raw Fastq files were quality filtered and clustered into 97% similarity operational classification units (OTUs). The OTU taxonomic annotation results are presented in Supplementary Material S6. The appropriate taxonomic level for microbiome analysis of the gut flora was selected based on these results.

### Analysis of community species diversity

Based on the findings of high-throughput sequencing, suitable classification levels were selected to study the diversity of gut microbiota in various groups with viral infections. The α-diversity (richness) of the gut microbiota at genus level in patients with different AGE viral infections was evaluated using the Chao1 index, and variations in species richness between different viral infection groups were investigated. Principal co-ordinates analysis (PCoA) was conducted to evaluate the β-diversity of the gut microbiota at genus level and analyze the variations in microbial species composition among different viral groups [41].

### Microbial correlation network analysis

The microbial correlation network analysis was applied to investigate the potential interactions of gut microbiota communities in patients with varying AGE virus infections [42,43]. Species whose abundance ranks within the top 0.1% at the genus level were selected to construct microbial correlation networks, with a *p*-value <0.05 and R^2^ > 0.6 indicating significant correlations [44]. The resulting network was visualized, and topological properties were calculated using Gephi 0.9.2 and further analyzed for topological parameters [45]. Based on the number of species nodes in the microbial correlation networks, the main species are selected, and the effects of the virus-mediated gut bacterial community on the clinical symptom of patients were studied using attributes such as the number of major species nodes and connectivity, in conjunction with the clinical symptom of patients with different viral infections.

### Key gut microbiota species identification

Through the linear discriminant analysis (LDA) and construction of random forest model (RF), the existence of key species in the gut microbiota of patients with different viral infections was investigated, with a focus on their influence the clinical symptom. The raw data obtained from sequencing were utilized to predict potential pathogenic bacteria using the 16sPIP established by the China CDC [46]. The outcomes from these methods were analyzed to identify key species in the gut microbiota.

### Statistical analysis

The Shapiro–Wilk test was used to verify the normality of the data. If the data conformed to a normal distribution, one-way ANOVA was utilized to compare among the three groups, and a t-test was utilized to compare between the two groups. If the data did not conform to a normal distribution, Kruskal–Wallis test was utilized to compare among the three groups, and Mann–Whitney U test was used to compare between the two groups. The analyses referenced above were performed by GraphPad Prism 5.01, and results are considered significant different at a *p* < 0.05. Random forest model and linear discriminate analysis were used to explore the differential species in the gut microbiota of patients infected with different AGE viruses. Microbial correlation networks analyses were performed to assess the impact of differential species on the course of clinical symptom. The analyses referenced above were performed using R 4.0.5.

### Ethics statement

The study was conducted in accordance with the Declaration of Helsinki, all subjects or their guardians were informed that the fecal samples would be used for AGE virus detection and possible high-throughput sequencing, and all personal privacy is guaranteed. Informed consent was obtained verbally from all subjects or guardian of subjects. The study and consent procedures were approved by the Medical Ethics Committee of Zhejiang Chinese Medical University (The ethical number is 20210201–1).

## Results

### Assessment of AGE patient clinical symptom by the new scoring system

Referring to the Vesikari scale of Freedman [38], and the reporting of Lai [39] and Zaraket [40], diarrhea, vomiting, abdominal pain, fever, fecal character, fecal occult blood, fecal leukocytes, white blood cell count, lymphocyte percentage and CRP were selected as indicators for clinical symptom scores. Due to the differences in each qualitative or quantitative clinical symptom, the assessment of clinical symptom was carried out by treating a normal or negative result as a score of 1. Dehydration is a major cause of life-threatening illness in patients with acute gastroenteritis, and vomiting and diarrhea are major factors in disrupting the hydration status of patients [20,47]. The greater the number of episodes of vomiting and diarrhea, the more dehydrated the patient is likely to be [47]. Therefore, 1 time of vomiting or diarrhea is recorded as 1 point, and so forth.

Lai et al. [39] found a significant association between symptoms, such as fecal occult blood, fecal leukocytes and CRP, and hospital admission in AGE patients. Referencing the studies of Lai [39] and Freedman [38] et al., symptoms, such as fecal character, fecal occult blood, fecal leucocyte, abdominal pain, and fever, were scored as 1 if negative, and 2 if positive. CRP, serving as a quantitative index, could accurately reflect the level of inflammation in patients and was categorized into four levels for the comparative scoring. Although white blood cell count and lymphocyte percentage were quantitative indicators, their responses to clinical symptom were multi-factorial, and scores were assigned based on a negative and positive scoring scale. The specific details of the scoring system are presented in Table 1. The distribution of clinical symptom scores of 289 patients using the scoring scale is presented in Table 2. The clinical symptom scores were 12.58 ± 1.27 in the None-virus group, 14.67 ± 1.58 in the Single-virus group, and 15.63 ± 1.30 in the Dual-virus group.

**Table 1.** Clinical symptom scoring scale for AGE patients.

Items	1 point	2 points	3 points	4 points or more
Fecal character	Mushy	Watery	/	/
Fecal occult blood	Negative	Positive	/	/
Fecal leucocyte	Negative	Positive	/	/
White blood cell count	≤10 × 10^9^/L	>10 × 10^9^/L	/	/
Lymphocytes%	≤50%	<50%	/	/
Abdominal pain	Negative	Positive	/	/
Fever	Normal	above 37.3°C	/	/
CRP	≤4.0 ng/mL	4.1–10 ng/mL	10.1–20 ng/mL	>20 ng/mL
Diarrhea	1 time	2 times	3 times	one point at a time
Vomiting	1 time	2 times	3 times	one point at a time

**Table 2.** Distribution of clinical symptom scores in 289 AGE patients.

Items	Clinical Symptom	None-virus	Single-virus	Dual-virus
Fecal character	Mushy	136	33	5
	Watery	84	27	3
Fecal occult blood	Negative	203	29	4
	Positive	18	31	4
Fecal leucocyte	Negative	211	43	6
	positive	10	17	2
White blood cell count	≤10 × 10^9^/L	157	33	5
	>10 × 10^9^/L	64	27	3
Lymphocytes%	≤50%	150	36	6
	>50%	71	24	2
Abdominal pain	Negative	142	35	5
	positive	79	25	3
Fever	Normal	191	36	5
	above37.3°C	30	24	3
CRP	≤4.0 ng/mL	149	29	2
	4.1–10 ng/mL	68	25	3
	10.1–20 ng/mL	3	2	1
	>20 ng/mL	1	4	2
Diarrhea	1 time	116	25	2
	2 times	101	28	5
	3 times	4	4	0
	one point at a time(≥4)	0	3	1
Vomiting	1 time	193	41	3
	2 times	28	19	4
	3 times	0	0	1
	one point at a time(≥4)	0	0	0
	average score	12.58 ± 1.27	14.67 ± 1.58	15.63 ± 1.30

### Comparison of clinical symptom scores in AGE patients

The clinical symptom of patients with viral AGE (clinical information sheet of Supplementary Material S1) and the clinical symptom of patients in the different viral infection groups were assessed according to the scoring scale in Table 1, and the results of this assessment were listed in scoring results sheet of Supplementary Material S1.

**Figure 1.**
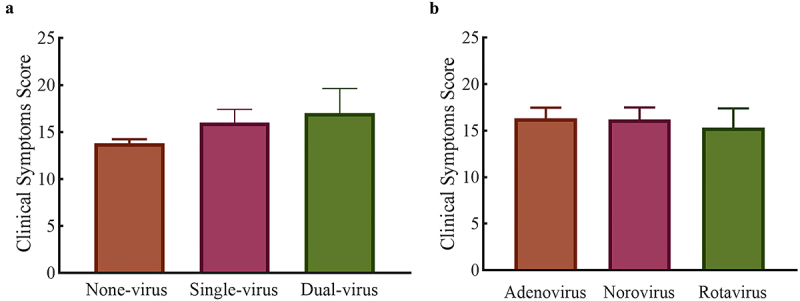
Comparison of clinical symptom assignment scores in AGE patients. (a) Grouping of the number of infected viruses, (b) grouping of the species of infected viruses.

The clinical symptom scores for the Single-virus, Dual-virus, Adenovirus, Norovirus, and Rotavirus groups are normally distributed, whereas those for None-virus group are not normally distributed. Kruskal–Wallis test indicated significant differences in clinical symptom scores among patients in the Single-virus, Dual-virus, and None-virus groups (*p* = 0.03) (Figure 1a). Specifically, Single-virus group ($\overset{ˉ}{x}$ = 14.82 ± 1.89) and Dual-virus group ($\overset{ˉ}{x}$ = 15.3 ± 2.08) exhibited more severe clinical symptom than the None-virus group ($\overset{ˉ}{x}$ = 12.4 ± 0.89). This indicates that viral infection exacerbated the clinical symptom in patients suffering from AGE. The Mann–Whitney U test revealed a significant difference in clinical symptom scores between None-virus group and Single-virus group (*p* = 0.02), while there were no significant differences between None-virus group and Dual-virus group, nor between Single-virus and Dual-virus group. This might be due to the small sample size of the Dual-virus group, which may not have been statistically significant. Further analysis using one-way ANOVA and unpaired t-test on the clinical symptom scores among the Adenovirus ($\overset{ˉ}{x}$ = 15.3 ± 2.08), Norovirus ($\overset{ˉ}{x}$ = 15.2 ± 1.48), and Rotavirus groups ($\overset{ˉ}{x}$ = 13.67 ± 2.52) showed no significant difference in the severity of clinical symptom among patients with these three distinct viral infections (Figure 1b). This finding suggested that infection with different types of viruses did not contribute to varying degrees of infection severity. In conclusion, the severity of clinical symptom in virus-infected patients was correlated with the presence or absence of AGE virus, but not with the virus species.

### AGE affected the taxonomic composition of gut communities

**Figure 2.**
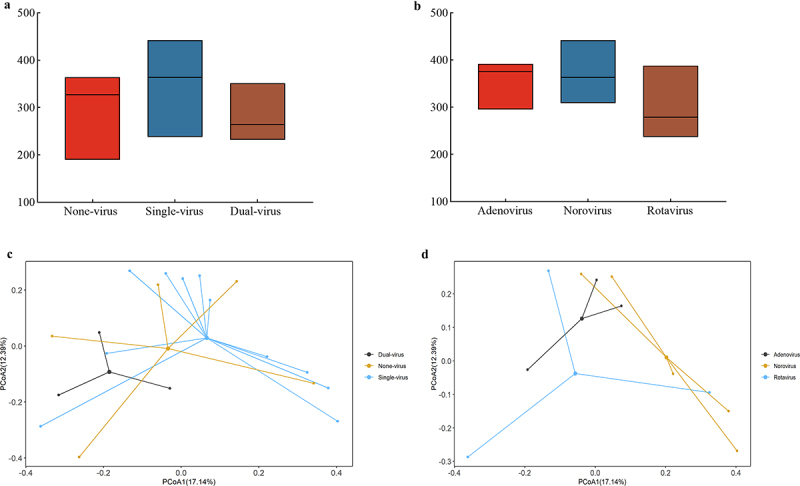
Community variation of gut microbiota in different ACE groups. Richness determined as Chao1 index per (a) patients with different number of virus types, and (b) patients with different kinds of virus, (c-d) compositional dissimilarity (genus level) of gut microbial communities per (c) patients with different number of virus types, and (d) patients with different kinds of virus.

Alpha diversity as richness was determined using the Chao1 index per sample (Table S7.1 of Supplement material S7). The Chao1 index of None-virus group was 287.40 ± 81.66, that of Single-virus group was 344.91 ± 59.80, and that of the Dual-virus group was 281.67 ± 62.14. A comparison of the Chao1index among these groups showed no significant differences, although the median value of the Single-virus group was relatively higher than the others (Figure 2(a)). The Chao1 index of Adenovirus group was 354.00 ± 51.80, that of Norovirus group was 365.60 ± 50.41, and that of the Rotavirus group was 301.33 ± 77.94, with no significant differences in chao1 indices among the groups (Figure 2(b)). Therefore, it can be deduced that no significant differences were detected in the richness of the gut microbial community between the groups. Principal Coordinate Analysis (PCoA) and the Adonis test (Figure 2(c)) indicated a significant distinction in genus composition between Single-virus and Dual-virus groups (Variation = 0.11, *p* = 0.034, as shown in Table S8.1 of Supplementary Material S8), suggesting that the number of viral infections influences the gut microbiota differently. In contrast, no notable differences were observed in the communities of patients infected with Adenovirus, the Norovirus, and the Rotavirus (Figure 2(d)). The chao1 index and PCoA analysis together revealed that multiple viral infections affect the gut microbial communities of patients by changing the species composition, rather than altering the richness of species.

### AGE virus infections promote the competitive interactions among the gut microbiota communities

**Figure 3.**
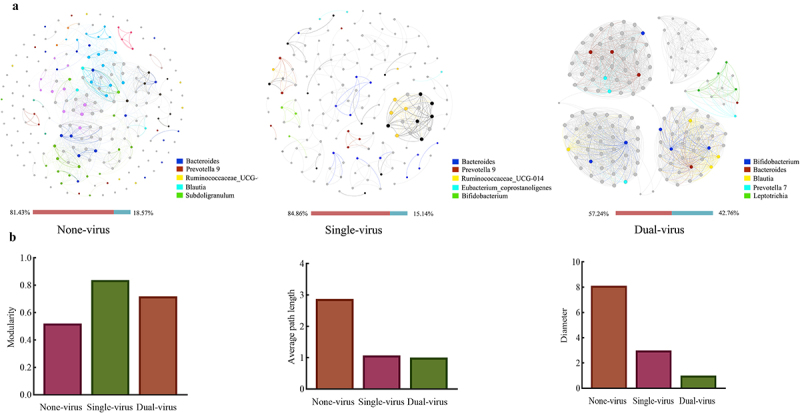
Construction of microbial correlation networks of gut microbiota in AGE patients. (a) Visualization of networks for three AGE groups (Brown: proportion of positive edges, blue: proportion of negative correlation edges); (b) topological parameters of the correlation networks, from left to right are modularity, average path length and diameter, respectively.

To further investigate the effect of multiple viral infections on microbial interactions within gut microbes, the microbial correlation networks were constructed. The proportion of positive correlation edges was 81.43% in None-virus group, 84.86% in Single-virus group, and 57.24% in the Dual-virus group, indicating a decrease in the proportion of positive correlation edges in the microbial correlation network in Dual-virus group compared to None-virus group (Figure 3(a)). This suggests that viral co-infections create a competitive environment within gut microbiota communities of patients. When comparing the topological properties of microbe-associated networks, the modularity was found to be higher in Single-virus (0.84) and Dual-virus group (0.72) compared to the None-virus group (0.52). The mean path lengths of Single-virus (1.00) and Dual-virus group (1.07) were smaller than those of None-virus group (2.87); and the diameters of in Single-virus (3.00) and Dual-virus group (1.01) were smaller than those of None-virus group (8.11) (Figure 3(b)). These findings indicated that an increase in the number of AGE virus infections may promote interactions among the gut microbiota communities of the patients.

Simultaneously, an analysis of the topological properties of the three microbial correlation networks of the Adenovirus, Norovirus, and Rotavirus groups was conducted (refer to Supplementary Material S9, Figure S9.1). The results showed that the Norovirus group had a longer mean path length and diamete, with a lower mean degree, while the Rotavirus group exhibited a lower proportion of positive correlation edges. This suggests that while the type of virus may have a minimal impact on patient clinical symptoms, it could exert a specific influence on the interaction within gut microbiota communities. In conclusion, infection with AGE virus appears to enhance interactions among gut microbiota communities in patients. An increase in the number of viral infections leads to a competitive relationship among gut microbiota, which may correlate with the severity of clinical symptom in AGE patients.

### The key gut microbiota species influenced by AGE virus infection are associated with the clinical symptoms

**Figure 4.**
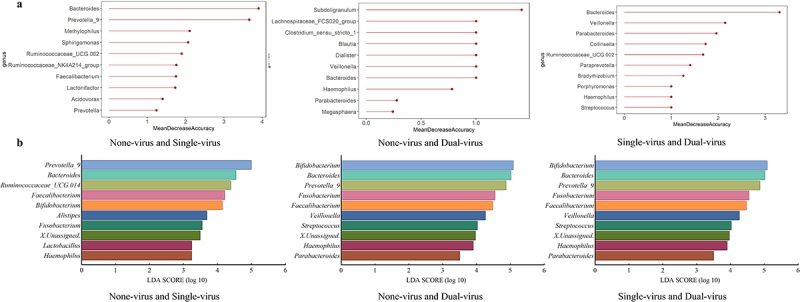
Genus analyzed for differences between gut microbiota of different viral infection groups (a) random forest model: (b) linear discriminate analysis.

By pairwise comparison of samples from None-virus, Single-virus, and Dual-virus group through the random forest model and LDA analysis (Figure 4), the top 10 species were selected for further analysis. It was discovered that *Bacteroides* ($\overset{ˉ}{x}$ = 4.86 ± 0.27), *Prevotella* ($\overset{ˉ}{x}$ = 4.92 ± 0.07), and *Bifidobacterium* ($\overset{ˉ}{x}$ = 4.77 ± 0.54) might be the key species influencing the clinical outcomes of patients. Additionally, the LDA results of Dual-virus and None-virus groups were extremely close to the LDA results of Dual-virus and Single-virus, suggesting that Dual-virus infections have a more significant impact on the gut microbial species, aligning with the findings of community variations (Figure 2(c)).

The raw data obtained from sequencing was further compared with the CDC 16sPIP to predict the potential disease-causing bacteria [46]. The top five possible pathogenic bacteria in each sample were screened out (Figure S10.1 of Supplementary Material S10). This result was consistent with the Random Forest and LDA analyses (Figure 4), revealing similarities between key species of the gut bacteria of patients with different types of viral infections and the predicted results of the 16sPIP. There were differences in the species composition of the gut microbiota among patients with different viral infections, and these distinct species might be the key factor in the varying severity of clinical symptom observed in patients.

### Viral infection leads to the imbalance of gut microbial community

An analysis of the nodes in the microbial molecular network of key gut microbiota species in patients with AGE virus revealed that the ratio of *Prevotella* degree to *Bacteroides* degree (P/B index) was significantly higher in Single-virus group (1.57) and Dua-virus group (1.28) compared to the None-virus group (0.10), with the former being over 10 times greater. Furthermore, the patients with a higher P/B ratio exhibited more severe clinical symptom, as indicated in Table 3 and Figure S10.2 of Supplementary Material S10. Comparison of the relative abundance ratios of *Prevotella* and *Bacteroides* across the groups also demonstrated consistency, with the Single-virus group (0.48) and the Dual-virus group (0.54) exhibiting higher ratios than the None-virus group (0.37). The result demonstrated a consistent pattern, indicating that an increase in *Prevotella* abundance could potentially contribute to inflammatory changes in the gut environment of patients. It was further surmised that viral infections impacted the competition between different species of gut bacteria in patients, resulting in an increased P/B index within the gut microbiota. The rise in the P/B index led to an imbalance in the gut microbial community, ultimately affecting the overall health of patients. Combined with microbial correlation network analysis (Figure 3), it was observed that the P/B index of the gut microbiota community of patients increased as the number of viral infections increased. Additionally, the worsening of gut microbiota interspecies competition exacerbated bacterial imbalance severity and further intensified the clinical symptom of patients.

**Table 3.** Correlation between *Prevotella*/*Bacteroides* index and clinical symptom scores.

P/B	Relative abundance ratio 95%Cl (0.29–0.63)	Degree ratio 95%Cl (−0.54–2.51)	Clinical symptom score 95%Cl (11.69–16.94)
None-virus	0.37	0.10	12.80
Single-virus	0.48	1.57	14.82
Dual-virus	0.54	1.28	15.33

## Discussion

### A well-established and systematic clinical symptom scoring scale assists in quantify the severity of clinical symptoms

Viral acute gastroenteritis (AGE) causes the variety of clinical symptom (i.e. diarrhoea, vomiting, fever, and abdominal pain) that typically manifest within 1–2 days of infection and persist for 3–8 days [48,49]. In this study, the clinical symptom scoring scale was used to compare the severity of clinical symptom among patients in the None-virus, Single-virus, and Dual-virus groups, revealing significantly difference. These findings were similar to those of Zaraket et al. [40]. No significant differences in clinical symptom were observed among Adenovirus, Norovirus, and Rotavirus groups. Plancarte et al. [50] reported higher symptom scores in patients with Rotavirus infection compared to those without viral infections, yet symptom scores did not differ among patients with other AGE viral infections patients, such as Norovirus. Hikita [48] compared the primary symptoms (fever, abdominal pain, vomiting, or diarrhea) and found a difference in fever and abdominal pain between Rotavirus and Norovirus infections. However, when compared using Vesikari scores, the difference was not statistically significant, which aligns with our results. Xie et al., Hikita et al., and Plancarte et al. [48,50,51] had selected similar AGE viruses for clinical symptom scoring and have reached conclusions that are partially variable, which may be related to sample volume and the type of clinical indicators collected. This suggests the need for a well-established and systematic clinical symptom scoring scale to score patients’ clinical symptoms. This will establish a quantitative link between gut bacterial microbiological analysis and clinical symptoms, which will help to investigate the correlation between gut microbiota and clinical symptoms in viral AGE patients.

The discrepancy in the results of symptom comparisons in different reports may be due to the lack of a longitudinal study in which multiple fecal samples and clinical information from the same patient over the course of the disease were collected, which is also a limitation of our study. In this study, our sample size was not large enough, but it was sufficient to support our study, and more samples will be collected subsequently to verify the reliability and validity of the clinical symptom scoring scale.

### New clinical symptom scores help to reveal virus-gut microbiota interaction

The analysis of the gut microbiota in AGE patients revealed significant differences in species composition between the Single-virus and Dual-virus groups (Figure 3, Supplementary Material Table S8.1). Additionally, viral infections were found to enhance competition within the gut microbiota of AGE patients, contributing to the exacerbation of clinical symptom. Ma et al. [52] and Quave et al. [53] also reported that AGE virus can affect gut microbiota by increasing opportunistic pathogens in patients suffering from viral AGE, thereby posing a potential threat to health of AGE patients. Ke et al. [54] observed a reduction in the stability of the gut microbiota community in their studies on inflammatory bowel disease, which was similar to our findings. Additionally, Kim et al. [55] reported that Rotavirus, Norovirus, Adenovirus, and Astrovirus could cause a particular alteration in the gut microbiota species following infection, thereby modifying the dominant gut microbiota and increasing the risk of pathogenic bacterial infection. The results in this study are similar, indicating that viral infections disrupt the gut microbiome’s homeostasis in patients, thereby exacerbating the clinical symptom. Although no significant differences in gut microbiota diversity and composition were observed among Norovirus, Rotavirus, and Adenovirus group in the study, Mathew et al. [56] reported that patients with Norovirus and Rotavirus infections showed significant differences in the gut microbiota diversity. By combining the clinical symptom scores of patients with the microbiological correlation networks for analysis (as shown in Table S9.1 of Supplementary Material S9), it was discovered that the P/B index was positively correlated with the clinical symptom scores of patients. Furthermore, changes in the P/B index denoted an elevation in the intestinal microenvironment inflammation levels of patients [57].

[58] Discovered that certain species in *Prevotella* and *Bacteroides* were linked to the emergence of IBS sequelae and symptoms resulting from AGE infections, and this was classified as a “microbial dysbiosis index.” This suggests that viral infection drives gut microbiota community competition. Additionally, with an increase in viral infections, with increased number of viruses infecting patients, the balance of the gut microbiota shifts, leading to a worsening in clinical symptom. However, in this study, no further distinction was made between the co-infections of different species of viruses, which were only categorized as Dual-virus group. Different species of viruses, due to differences in their mechanisms of infection, may have different impacts on the intestinal environment of patients during co-infections, which may further affect the gut microbiota and the health of the patients.

### Gut microbiota key species have potential impact on patient clinical symptoms

In this study, the AGE virus was found to impacts the species composition of the gut microbiota, promoting competition and resulting in an imbalanced bacterial community and worsening clinical symptom in patients [55]. Further analyses by RF and LDA revealed that *Bacteroides*, *Prevotella,* and *Bifidobacterium* might be the key species. *Bacteroides* and *Prevotella* are considered the cornerstone species of the human gut microbiota [59,60], playing a crucial role in maintaining the stability of the gut microbiota. Changes in the P/B index after viral infection can trigger inter-bacterial responses that have implications for patient health [61].

Through analysis of microbial correlation networks major node species, we can speculate that in None-virus group, patients exhibited the lowest clinical symptom score and a subdued inflammatory response in their intestinal environment. This may be attributed to the probiotic effects of the microbial related network node species. Especially, *Subdoligranulum* particles can counteract the inflammatory environment of the intestine [62], *Blautia* eased inflammatory diseases and reduced intestinal inflammation, and *Ruminococcaceae_UCG-014* supported intestinal health [63,64]. In the Single-virus group, patients had the second-highest clinical symptom score, potentially because of the presence of two probiotics, *Ruminococcaceae_UCG-014* and *Bifidobacterium,* which have a protective effect on the intestinal microenvironment [63,65]. In the Dual-virus group, despite the presence of probiotics such as *Bifidobacterium*, *Bacteroides*, and *Blautia* among the key node species, these species had a higher degree of connectivity compared to the average degree of the gut microbiota. This suggests that the virus may have intensified competition for probiotics, resulting in the highest clinical symptom scores for their patients. The role of bacteria extends beyond a mere relationship of competition or cooperation, it is more likely to be a long-term dynamic influence [61,66].

Depending on the specific viral infection, the gut microbiota community can consist of partially identical and individually specific species, which further influence the clinical symptom [67] (see Table S10.1 in Supplementary Material S10). While viral infections increase the severity of patient symptoms, certain bacteria may counter this response, presenting new avenues for rational prevention and treatment of AGE viruses [68], modifying or transplanting the gut microbiota could have therapeutic effects on intestinal inflammatory or viral infections. It may be feasible to identify and isolate bacteria with antagonistic effects on viruses and develop them into a probiotic treatment for a particular AGE virus. In the study, the interference of common clinical AGE pathogenic bacteria was excluded. However, the absence of normal, healthy human gut microbiota as a control in the study may have influenced the identification of key species.

## Conclusions

The clinical symptom of viral acute gastroenteritis (AGE) patients was evaluated using a clinical symptom scoring scale to establish a correlation between symptom severity and gut microbiota. The study revealed that the type of AGE virus infection had little effect on clinical symptom, and the quantity of AGE viruses promoted the competitive environment for the gut microbiota of AGE patients, which can exacerbate the severity of these symptoms.

## Supplementary Material

Clean copy of supplementary material 2- QVIR-2024-0335.R1 .docx

Supplementary Material S6.xlsx

Supplement Materials S5.docx

FigureS10_2.jpeg

Supplement Materials S10.docx

Supplement Materials S9.docx

Supplement Materials S3.docx

Supplementary Material S1.xlsx

Supplement Materials S7.docx

FigureS10_1.jpeg

Supplement Materials S8.docx

FigureS9_1.jpeg

Supplement Materials S4.docx

## Data Availability

The data generated during the study and supplementary materials are available at repository name Science Data Bank at https://doi.org/10.57760/sciencedb.10757.
