# Supplementary material for: Impact of virus-mediated bacterial interactions on acute gastroenteritis symptoms: A new scoring system for clinical assessment
Source: Virulence. 2025 Jul 7;16(1):2529442. doi: 10.1080/21505594.2025.2529442 (PMC12269689; doi:10.1080/21505594.2025.2529442)
Supplement: Clean copy of supplementary material 2- QVIR-2024-0335.R1 .docx [file KVIR_A_2529442_SM1901.docx]

Supplement material S2: General Characteristics of AGE patients

Between February 2021 and January 2022, a total of 289 stool samples from AGE patients without fungal, parasitic and bacterial infections (i.e. *Salmonella sp*., *Shigella sp.*, *Vibrio cholerae*, *Vibrio parahaemolyticus* and Diarrheogenic *Escherichia coli*) was available for analysis. A total of 154 (53.29%) of the patients were male and the median age was 6 years with the youngest individual being less than 6 months and the oldest 92 years.

Table S2.1 Demographic and samples characteristics of patients

| Characteristic | Number |
| --- | --- |
| Patients | 289 (100.00%) |
| Male | 154 (53.29%) |
| Children (＜18 years) | 179 (61.94%) |
| AGE virus detection | 68 (23.53%) |
