# Supplementary material for: Impact of virus-mediated bacterial interactions on acute gastroenteritis symptoms: A new scoring system for clinical assessment
Source: Virulence. 2025 Jul 7;16(1):2529442. doi: 10.1080/21505594.2025.2529442 (PMC12269689; doi:10.1080/21505594.2025.2529442)
Supplement: Supplement Materials S5.docx [file KVIR_A_2529442_SM1899.docx]

Supplement material S5: General Characteristics of high throughput sequencing

Based on the detection results of AGE virus, 19 fecal samples were selected and sent to Guangzhou Magigene Biological Technology Co. Ltd. High-throughput sequencing was performed based on the bacterial 16S rRNA coding gene (V4 variable region, 515F-806R), and the Raw Fastq files obtained by sequencing were quality filtered and aggregated to form OTUs with 97% similarity. A total of 1.51×10^6^ high-quality sequence numbers were obtained, and the final classification annotation 3293 OTUs were obtained, and the sequence numbers and OTU numbers of each AGE virus grouping are shown in Table S5.1. Sequencing data were available in a text file named “Supplementary Material S6.txt”.

Table S5.1 Basic information about high-throughput sequencing

| Group | Number | OTU | Sequence reads |
| --- | --- | --- | --- |
| Norovirus | 5 | 852 | 408969 |
| Rotavirus | 3 | 567 | 241375 |
| Adenovirus | 3 | 633 | 227360 |
| Dual-virus | 3 | 528 | 237196 |
| None-virus | 5 | 713 | 398993 |

PS: Three groups of Norovirus, Rotavirus and Adenovirus form the Single-virus group
