# Supplementary figures and images for: Impact of virus-mediated bacterial interactions on acute gastroenteritis symptoms: A new scoring system for clinical assessment
Source: Virulence. 2025 Jul 7;16(1):2529442. doi: 10.1080/21505594.2025.2529442 (PMC12269689; doi:10.1080/21505594.2025.2529442)

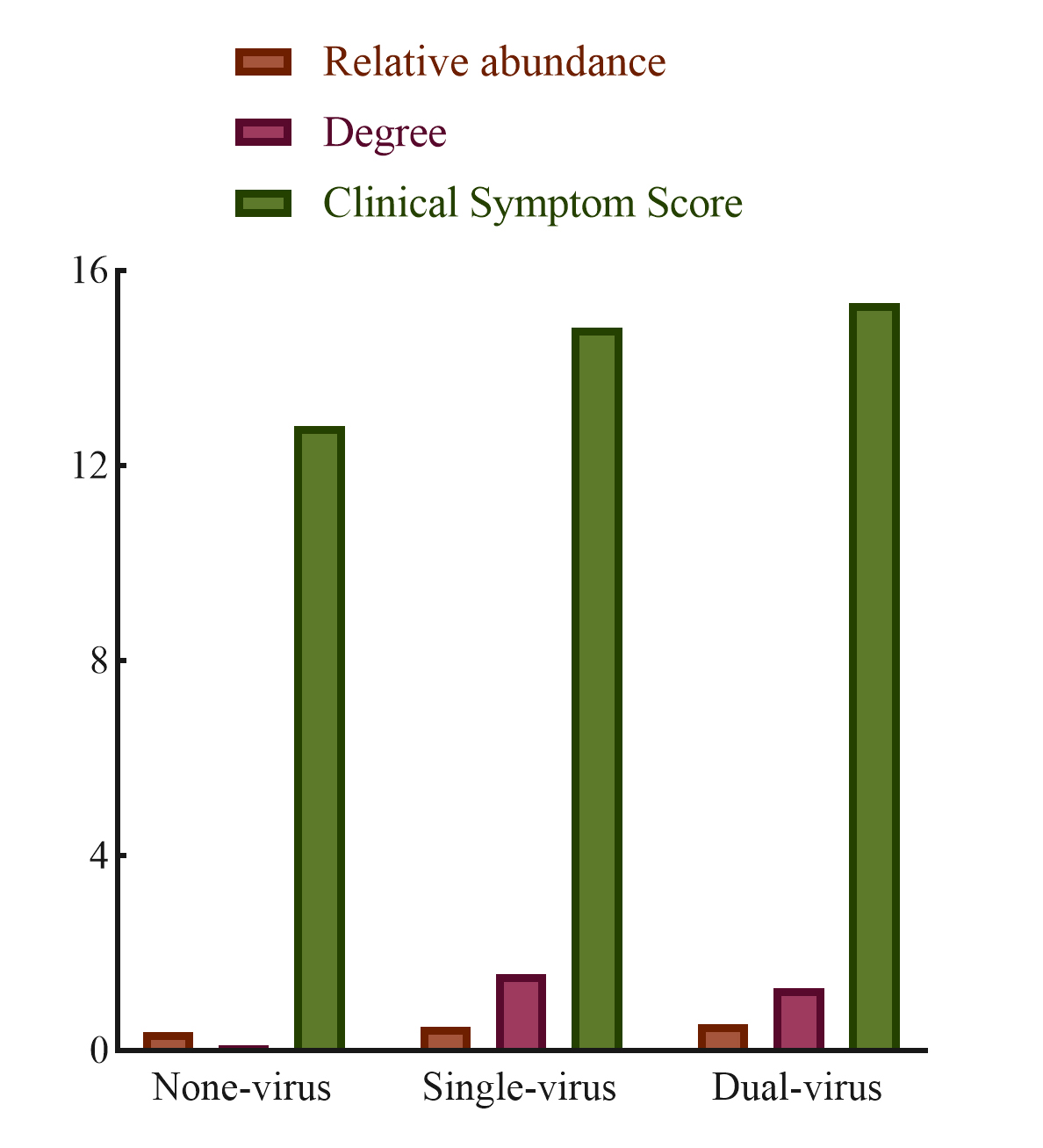

Supplement: FigureS10_2.jpeg [file KVIR_A_2529442_SM1898.jpeg]
