# Supplementary material for: Impact of virus-mediated bacterial interactions on acute gastroenteritis symptoms: A new scoring system for clinical assessment
Source: Virulence. 2025 Jul 7;16(1):2529442. doi: 10.1080/21505594.2025.2529442 (PMC12269689; doi:10.1080/21505594.2025.2529442)
Supplement: Supplement Materials S10.docx [file KVIR_A_2529442_SM1897.docx]

Supplementary Material S10：Prediction of pathogenic bacteria on the 16sPIP

The raw data obtained from the sequencing were compared with the 16sPIP platform of the China CDC to predict the bacteria that may cause disease. The top five most likely pathogenic bacteria in each sample were screened out. This was corroborated by the Random Forest and LDA analyses.


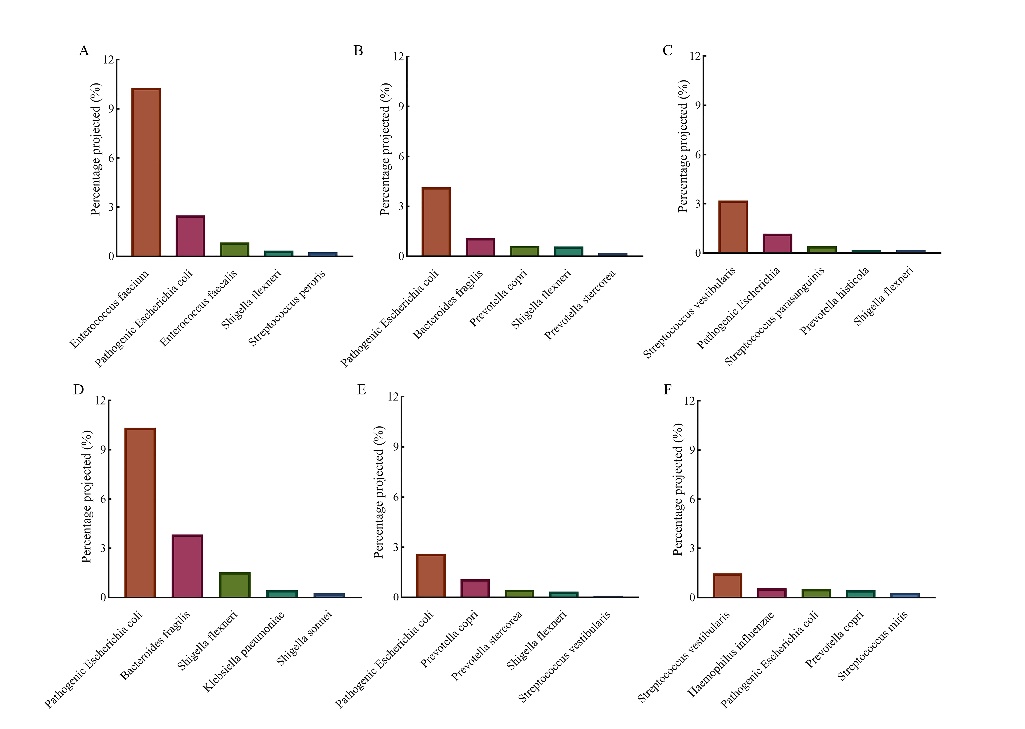


FigureS10.1 Prediction of pathogenic bacteria on the 16sPIP for each group of gut bacterial communities

A、None-virus, B、Single-virus, C、Dual-virus, D、Adenovirus, E、Norovirus, F、Rotavirus

Table S10.1 Possible presence of pathogenic bacteria in different groups of gut microbiota and their clinical significance

| Species | Significance | Group |
| --- | --- | --- |
| *Bacteroides fragilis* | Associated with chronic tissue inflammation and the release of pro-inflammatory | Single-virus |
|  | Up-regulating anti-inflammatory factor IL-10 |  |
|  | Associated with abdominal pain in IBS |  |
| *Prevotella copri* | Resulted in inflammation and immune dysregulation |  |
|  | Associated with diarrhea during the travel |  |
|  | Linked with chronic inflammatory conditions |  |
| *Streptococcus mitis* | Associated with moderate to severe diarrhea | None-virus |
| Pathogenic *Escherichia coli* | Diarrhea-associated bacteria | Single-virus、Dual-virus |
|  | Associated with enteric bacterial infections |  |


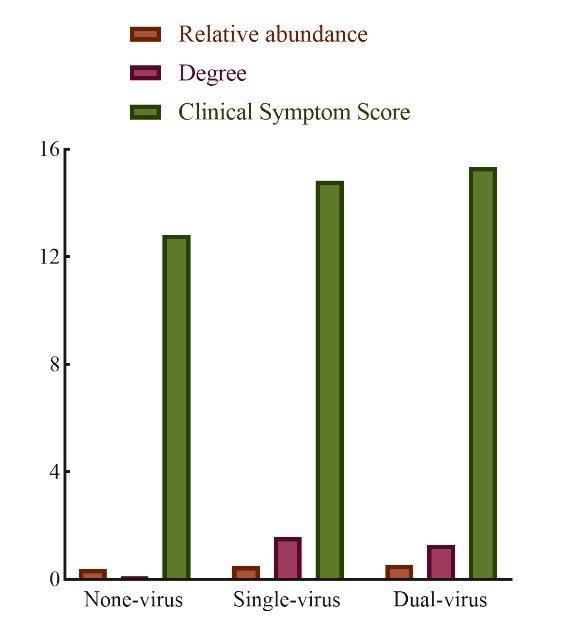


Figure S10.2 Correlation of P/B Index with Clinical Symptom Scores
