# Supplementary material for: Impact of virus-mediated bacterial interactions on acute gastroenteritis symptoms: A new scoring system for clinical assessment
Source: Virulence. 2025 Jul 7;16(1):2529442. doi: 10.1080/21505594.2025.2529442 (PMC12269689; doi:10.1080/21505594.2025.2529442)
Supplement: Supplement Materials S9.docx [file KVIR_A_2529442_SM1896.docx]

Supplementary Material S9：Construction of different microbial correlation networks and analysis of main nodes species attributes


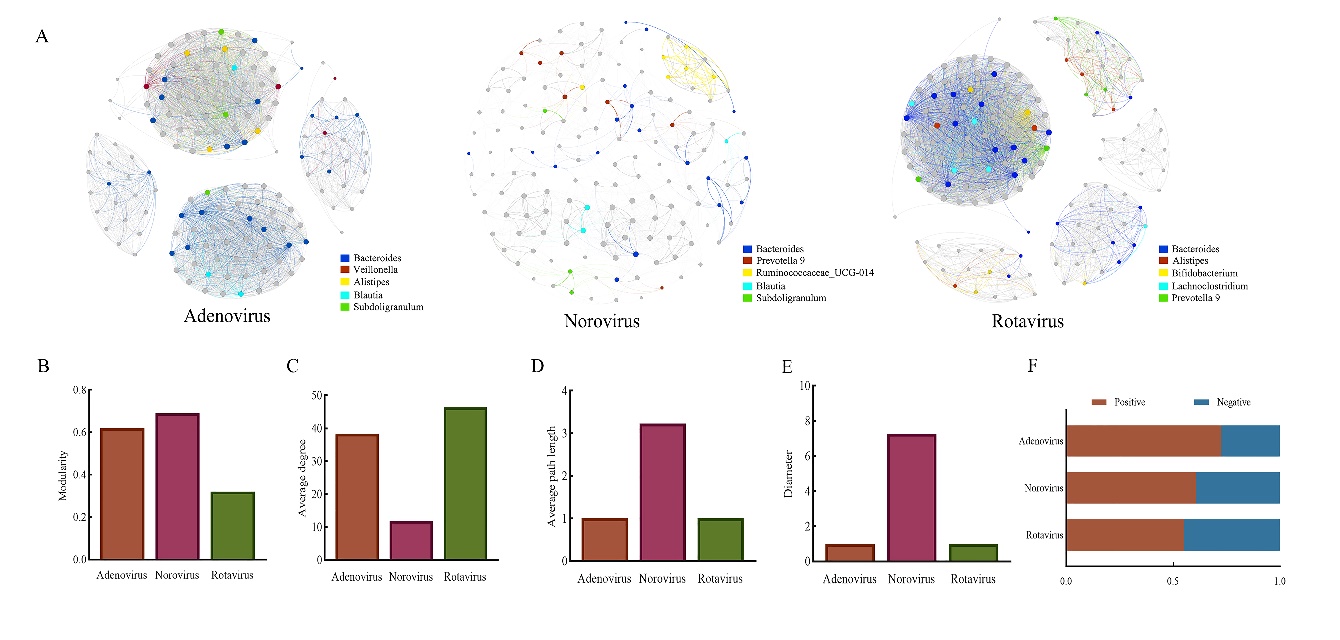


Figure S9.1 Construction of microbial correlation networks of gut microbiota in different AGE virus

B、Modularity; C、Average path length; D、Average degree; E、Diameter; F、Proportion of positively correlated sides

Table S9.1 Species of the main nodes of the microbial correlation networks and average degree

| Group | Genus | Nodes | Proportion | Average degree | Average degree of all nodes |
| --- | --- | --- | --- | --- | --- |
| None-virus | *Bacteroides* | 18 | 0.12 | 12.56 | 15.54 |
|  | *Prevotella* | 7 | 0.0467 | 1.29 |  |
|  | *Ruminococcaceae_UCG-014* | 6 | 0.04 | 0 |  |
|  | *Blautia* | 3 | 0.02 | 20.67 |  |
|  | *Subdoligranulum* | 3 | 0.02 | 16.67 |  |
| Single virus | *Bacteroides* | 13 | 0.0876 | 16.31 | 20.8 |
|  | *Prevotella_9* | 7 | 0.0511 | 25.57 |  |
|  | *Ruminococcaceae_UCG-014* | 6 | 0.0438 | 31.43 |  |
|  | *Bifidobacterium* | 3 | 0.0219 | 13.33 |  |
|  | *Dialister* | 3 | 0.0219 | 19.67 |  |
| Dual-virus | *Bifidobacterium* | 6 | 0.04 | 36 | 32.08 |
|  | *Bacteroides* | 5 | 0.0333 | 33.4 |  |
|  | *Blautia* | 4 | 0.0267 | 34.25 |  |
|  | *Prevotella* | 4 | 0.0267 | 42.75 |  |
|  | *Leptotrichia* | 4 | 0.0267 | 11 |  |
| Adenovirus | *Bacteroides* | 23 | 0.1533 | 38.04 | 38.36 |
|  | *Veillonella* | 4 | 0.0267 | 31.5 |  |
|  | *Alistipes* | 4 | 0.0267 | 52 |  |
|  | *Blautia* | 3 | 0.02 | 48 |  |
|  | *Subdoligranulum* | 3 | 0.02 | 50 |  |
| Norovirus | *Bacteroides* | 18 | 0.12 | 8.56 | 11.84 |
|  | *Prevotella* | 7 | 0.0467 | 11 |  |
|  | *Ruminococcaceae_UCG-014* | 6 | 0.04 | 10.33 |  |
|  | *Blautia* | 3 | 0.02 | 17 |  |
|  | *Subdoligranulum* | 3 | 0.02 | 10.67 |  |
| Rotavirus | *Bacteroides* | 22 | 0.1467 | 49.09 | 46.39 |
|  | *Alistipes* | 6 | 0.04 | 36.67 |  |
|  | *Bifidobacterium* | 5 | 0.0333 | 40.8 |  |
|  | *Lachnoclostridium* | 5 | 0.0333 | 64.4 |  |
|  | *Prevotella* | 5 | 0.0333 | 40.8 |  |
