# Supplementary material for: Impact of virus-mediated bacterial interactions on acute gastroenteritis symptoms: A new scoring system for clinical assessment
Source: Virulence. 2025 Jul 7;16(1):2529442. doi: 10.1080/21505594.2025.2529442 (PMC12269689; doi:10.1080/21505594.2025.2529442)
Supplement: Supplement Materials S3.docx [file KVIR_A_2529442_SM1895.docx]

Supplement material S3: The qPCR assay for AGE virus detection

The qPCR assay was used for virus detection, and five sets of primers for specific AGE viruses were shown in Table S3.1, and he mixture for qPCR assay mixtures was prepared as follows in Table S3.2, S3.3.

Table S3.1 Specific primers and probes for AGE virus

| Virus | | Primer and probe sequence (5'-3') | product size |
| --- | --- | --- | --- |
| NoV GI | F | GCCATGTTCCGITGGATG | 96 bp |
|  | R | TCCTTAGACGCCATCATCAT |  |
|  | P | HEX-AGATYGCGRTCTCCTGTCCACA-BHQ |  |
| NoV GII | F | CAAGAGTCAATGTTTAGGTGGATGAG | 89 bp |
|  | R | TCGACGCCATCTTCATTCACA |  |
|  | P | FAM-TGGGAGGGCGATCGCAATCT-TAMRAR |  |
| RoV A | F | ATGGATGTCCTGTACTCCTTGTCAAAA |  |
|  | R | TTCCTCCAGTTTGRAASTCATTTCC | 128 bp |
|  | P | FAM-AATGTACCTTCAACAATYTTRTCCCTAGC-TAMRA |  |
| AdV | F | GCCCCAGTGGTCTTACATGCACATC |  |
|  | R | GCCACGGTGGGGTTTCTAAACTT | 132 bp |
|  | P | HEX-TGCACCAGACCCGGGCTCAGGTACTCCGA-BHQ |  |

Table S3.2 The RT-qPCR assay mixtures for Norovirus

| Reaction reagent | Volume (μL) |
| --- | --- |
| 2× PCR reaction master mix | 12.5 |
| Taq enzyme | 0.5 |
| Reverse transcriptase | 0.5 |
| Forward Primer (20 μM) | 0.6 for each virus |
| Reverse Primer (20 μM) | 0.6 for each virus |
| Probe (20 μM) | 0.3 for each virus |
| Template | 5.0 |
| dd H_2_O | Add total volume up to 25 |

Multiplex real-time fluorescent quantitative polymerase chain reaction (Multiplex RT-qPCR) assay was modified based on the One Step Primescript^TM^ RT-PCR Kit instructions (Takara, Japan), the mixture for RT-qPCR assay mixtures was prepared as follows in Table3.3.

Table S3.3 The RT-qPCR assay mixtures for Rotavirus and Adenovirus

| Reaction reagent | Volume (μL) |
| --- | --- |
| 2× PCR reaction master mix | 12.5 |
| Taq enzyme | 0.5 |
| Reverse transcriptase | 0.5 |
| Forward Primer (20 μM) | 0.6 for each virus |
| Reverse Primer (20 μM) | 0.6 for each virus |
| Probe (20 μM) | 0.3 for each virus |
| Template | 5.0 |
| dd H_2_O | Add total volume up to 25 |

Multiplex RT-qPCR was carried out in the ABI 7500 Fast Real-Time PCR instrument (Applied Biosystems, US) under the following conditions: NoV GI, NoV GII: starting with reverse transcription at 42℃ for 30 min, followed by denaturation at 95℃ for 5 min, and 40 cycles of amplification (95℃ for 5 s and 55℃ for 55 s); RoV and AdV: reverse transcription at 42℃ for 30 min, denaturation at 95℃ for 5 min, and 40 cycles of amplification (95℃ for 5 s and 60℃ for 55 s). The cycle threshold (CT) was determined by the instrument. The RT-qPCR result was considered positive if amplification was detected within 40 cycles.
