# Supplementary material for: Impact of virus-mediated bacterial interactions on acute gastroenteritis symptoms: A new scoring system for clinical assessment
Source: Virulence. 2025 Jul 7;16(1):2529442. doi: 10.1080/21505594.2025.2529442 (PMC12269689; doi:10.1080/21505594.2025.2529442)
Supplement: Supplement Materials S7.docx [file KVIR_A_2529442_SM1893.docx]

Supplement material S7: Chao1 index per sample

Table S7.1 Chao1 index per sample

| Sample | chao1 | Group |
| --- | --- | --- |
| Norovirus | 335 | Single-virus |
| Norovirus | 442 | Single-virus |
| Norovirus | 309 | Single-virus |
| Norovirus | 379 | Single-virus |
| Norovirus | 363 | Single-virus |
| Rotavirus | 237 | Single-virus |
| Rotavirus | 279 | Single-virus |
| Rotavirus | 388 | Single-virus |
| Adenovirus | 375 | Single-virus |
| Adenovirus | 295 | Single-virus |
| Adenovirus | 392 | Single-virus |
| Dual-virus | 231 | Dual-virus |
| Dual-virus | 351 | Dual-virus |
| Dual-virus | 263 | Dual-virus |
| None-virus | 363 | None-virus |
| None-virus | 189 | None-virus |
| None-virus | 210 | None-virus |
| None-virus | 326 | None-virus |
| None-virus | 349 | None-virus |
