# Supplementary material for: Impact of virus-mediated bacterial interactions on acute gastroenteritis symptoms: A new scoring system for clinical assessment
Source: Virulence. 2025 Jul 7;16(1):2529442. doi: 10.1080/21505594.2025.2529442 (PMC12269689; doi:10.1080/21505594.2025.2529442)
Supplement: Supplement Materials S8.docx [file KVIR_A_2529442_SM1891.docx]

Supplement material S8: Adonis test for different viral infection groups

Table S8.1 Adonis test for different viral infection groups

| Group | Df | Mean squares | F.Model | Variation (R2) | Pr (>F) |
| --- | --- | --- | --- | --- | --- |
| Single-virus/Dual-virus | 1 | 0.525598 | 1.545055 | 0.114068 | 0.034 |
| Single-virus/None-virus | 1 | 0.26051 | 0.727218 | 0.049379 | 0.867 |
| Dual-virus/None-virus | 1 | 0.411484 | 1.023214 | 0.14569 | 0.435 |
| Norovirus/Rotavirus | 1 | 0.332882 | 1.023924 | 0.145777 | 0.324 |
| Norovirus/Adenovirus | 1 | 0.385871 | 1.280649 | 0.175898 | 0.261 |
| Rotavirus/Adenovirus | 1 | 0.36308 | 0.978347 | 0.19652 | 0.6 |
