# Supplementary material for: Impact of virus-mediated bacterial interactions on acute gastroenteritis symptoms: A new scoring system for clinical assessment
Source: Virulence. 2025 Jul 7;16(1):2529442. doi: 10.1080/21505594.2025.2529442 (PMC12269689; doi:10.1080/21505594.2025.2529442)
Supplement: Supplement Materials S4.docx [file KVIR_A_2529442_SM1889.docx]

Supplement material S4: AGE virus detection results by RT-qPCR

The 289 stool samples collected were detected for AGE virus by RT-qPCR, and 68 samples were positive. Among them, NoV GI was detected in 2 cases, with a positive rate of 0.69%; NoV GII was detected in 37 cases, with a positive rate of 12.80%; RoV was detected in 15 cases, with a positive rate of 5.19%; HAdV was detected in 22 cases, with a positive rate of 7.61%; HAV was not detected; the total detection rate was 23.53%, as shown in Table S4.1.

Table S4.1 Results of detection of AGE viruses in stool samples

| Virus | Positive samples | Positive rate |
| --- | --- | --- |
| NoV GI | 2 | 0.69% |
| NoV GII | 37 | 12.80% |
| HAdV | 22 | 7.61% |
| RoV | 15 | 5.19% |

Among the 68 positive samples, two AGE viruses were detected simultaneously in 8 samples, including 1 case of NoV GII and RoV, 4 cases of RoV and HAdV, and 3 cases of NoV GII and HAdV, as shown in Table S4.2.

Table S4.2 Results of simultaneous detection of AGE virus in stool samples

| Virus | Number |
| --- | --- |
| NoV GII+RoV | 1 |
| RoV+HAdV | 4 |
| NoV GII+HAdV | 3 |
